# Accelerated First-Principles Calculations Based on Machine Learning for Interfacial Modification Element Screening of SiCp/Al Composites

**DOI:** 10.3390/ma17061322

**Published:** 2024-03-13

**Authors:** Xiaoshuang Du, Nan Qu, Xuexi Zhang, Jiaying Chen, Puchang Cui, Jingtao Huang, Yong Liu, Jingchuan Zhu

**Affiliations:** 1 School of Materials Science and Engineering, Harbin Institute of Technology, Harbin 150001, Chinaqunan@hit.edu.cn (N.Q.); xxzhang@hit.edu.cn (X.Z.); 20b909032@stu.hit.edu.cn (J.H.); 2 National Key Laboratory for Precision Hot Processing of Metals, Harbin Institute of Technology, Harbin 150001, China

**Keywords:** SiCp/Al matrix composites, machine learning, first principle, interface modification element

## Abstract

SiCp/Al composites offer the advantages of lightweight construction, high strength, and corrosion resistance, rendering them extensively applicable across various domains such as aerospace and precision instrumentation. Nonetheless, the interfacial reaction between SiC and Al under high temperatures leads to degradation in material properties. In this study, the interface segregation energy and interface binding energy subsequent to the inclusion of alloying elements were computed through a first-principle methodology, serving as a dataset for machine learning. Feature descriptors for machine learning undergo refinement via feature engineering. Leveraging the theory of machine-learning-accelerated first-principle computation, six machine learning models—RBF, SVM, BPNN, ENS, ANN, and RF—were developed to train the dataset, with the ANN model selected based on R^2^ and MSE metrics. Through this model, the accelerated computation of interface segregation energy and interface binding energy was achieved for 89 elements. The results indicate that elements including B, Si, Fe, Co, Ni, Cu, Zn, Ga, and Ge exhibit dual functionality, inhibiting interfacial reactions while bolstering interfacial binding. Furthermore, the atomic-scale mechanism elucidates the interfacial modulation of these elements. This investigation furnishes a theoretical framework for the compositional design of SiCp/Al composites.

## 1. Introduction

Good interfacial bonding is an important guarantee to improve the properties of metal matrix composites, and the degree of interfacial reaction directly affects the strength of interfacial bonding [1]. Among these composites, SiCp/Al composite materials have good performance in terms of high-temperature resistance, corrosion resistance and high specific stiffness [2,3,4,5,6]. However, there is a serious interfacial reaction at the SiCp/Al interface, and the generated Al_4_C_3_ can seriously reduce the strength, elastic modulus, and corrosion resistance of aluminum matrix composites [7]. In addition to the fact that the interface reaction generated at the SiCp/Al interface will reduce the properties of the material, the degree of bonding between the SiCp/Al interface also has a great impact on the mechanical properties of the composite [8,9]. Existing studies have shown that the addition of alloying elements can not only inhibit the SiCp/Al interface reaction to a certain extent but also improve the bonding ability between the interfaces so as to improve the properties of composite materials [10,11]. For example, the addition of Si, Cu, Ti, etc. can inhibit the generation of Al_4_C_3_ and improve the mechanical properties of the material by reducing the activity or generating other substances [12,13,14]. However, there are still some deficiencies in the alloying elements added in the current study, such as the lack of explanation based on the atomic perspective for the mechanism of inhibiting the interfacial reaction through the addition of Si atoms in the matrix [15,16]; Cu atoms can form a solid solution with Al to improve the properties of the material, but the effect of their enhancement of the properties of the material is not significant enough, and the introduction of Ti is prone to generate Al-Ti brittle intermetallic compounds with Al, which leads to a reduction in the strength of the material. The introduction of Ti can easily generate Al-Ti brittle intermetallic compounds with Al, resulting in the reduction of material strength [17]. Therefore, it is necessary to expand the research scope of alloying elements and search for more suitable alloying elements within the scope of the periodic table.

Due to the complexity of the interface structure, mechanical deformation, and failure mechanism, it is difficult for traditional experimental methods to reveal the mechanism of interface bonding and segregation from the atomic point of view [18]. Therefore, most of the existing studies start from the first-principles calculation, apply the theory of quantum mechanics, calculate with the help of basic constants and reasonable approximations, and determine the state of the system according to the relationship between the total energy obtained and the electronic structure and the nucleus [19,20,21]. Although the first-principles calculation can explain the interface reaction from the atomic point of view, the calculation process needs a lot of resources and time. Therefore, the research on SiCp/Al interface reaction has been limited to a certain extent, and the composition design in the current research is mostly limited to a few common alloying elements. With the rapid development of material genetics and big-data technology, the emergence of machine learning provides a shortcut to solving the problems existing in first-principle computing. The machine learning dataset is obtained by sorting out the first-principles calculation results, using the machine learning model to train the dataset, and predicting the target through the trained model, which can realize the acceleration of the first-principles calculation via machine learning. For example, Miyazato et al. [22] used machine learning to accelerate first-principles calculations to predict the magnetic moments of two-hundred-and-fifty-four 2D materials and discovered eight stable 2D materials with high magnetic moments; Artrith et al. [23] utilized a machine learning method combining a genetic algorithm (GA) and artificial neural network (ANN) to accelerate the first-principles sampling of complex structural spaces of amorphous and disordered materials.

Although first-principles calculations are time-consuming and costly, they can be successfully solved with the acceleration of machine learning. Therefore, this paper combines two materials research methods, machine learning and first-principles calculations, to calculate the interface segregation energy and interface binding energy for elements in the periodic table. The screening of most of the elements in the periodic table was realized via machine-learning-accelerated first-principles calculations. We first calculated the interface segregation energy and interface binding energy of the SiCp/Al interfacial model when the model was doped with 25 different alloying elements using first principles. Then, the elemental properties that were most suitable for the interface segregation energy and interface binding energy were screened as input feature descriptors through feature engineering to form a complete dataset, and the model with the best performance was screened according to the R and MSE values. Finally, the prediction of interface segregation energy and interface binding energy for the remaining 89 elements was realized by accelerating the first-principles calculation through machine learning. Based on the prediction results, the elements that were prone to interfacial cohesion and the elements that enhanced interfacial bonding were selected. By taking the intersection of the two selected alloying elements, the alloying elements that inhibit interfacial reactions while also enhancing interfacial bonding were obtained. In this study, the machine learning method was used to break through the difficulty of the long computation time of the first principle and also to realize the screening of alloying elements for the whole periodic table except for some elements. The results of the study can greatly accelerate the matrix composition design of SiCp/Al composites.

## 2. Methods

### 2.1. First-Principles Calculations

**Figure A1:**
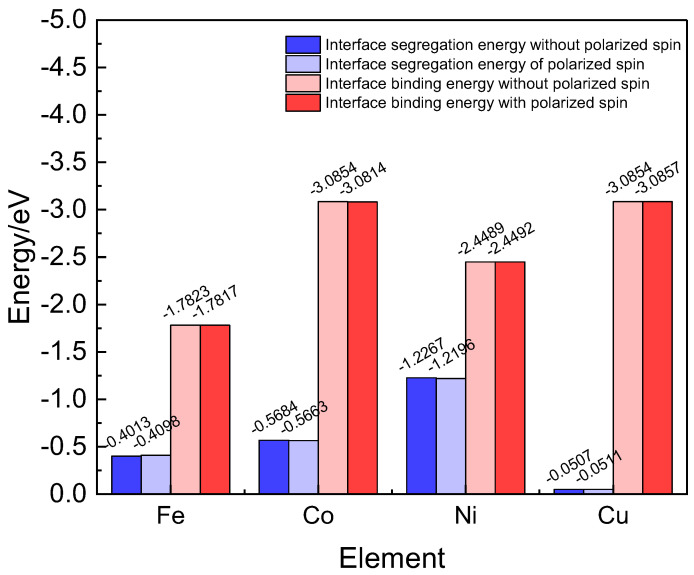
Energy of the system with the addition of magnetic alloying elements.

All the first-principles calculations in this experiment were based on the DFT and were performed using the CASTEP (Cambridge Serial Total Energy Package) module in the Materials Studio 8.0 software. All the energy calculations and the optimization process of the geometries in the paper use the PBE (Perdew–Burke–Ernzerhof) potential function under the Generalized Gradient Approximation (GGA) to describe the correlation interactions, solving the Kohn–Sham (KS) equations in a self-consistent way and using the ultra-soft pseudo-potential to describe the valence-electron interactions with ions [24]. With the help of computational methods, the truncation energy was determined to be 350 eV, and the Brillouin zone was sampled with a Monkhorst–Pack k-point grid, with the k-points taking the value of 6 × 6 × 6. During the geometry optimization process, it was determined that the force of chirp to each atom was not greater than 0.3 eV/nm and that the maximal distance of the atoms’ movement was 1 × 10^−4^ nm [25]. As shown in Figure A1, through calculation, it was found that spin polarization has little effect on the interface segregation energy and interface binding energy of the model, so the results in this paper do not involve the spin polarization of magnetic alloy elements. In order to obtain accurate energy calculations and the interface binding energy and interface segregation energy, the geometry of the interfacial model is optimized before calculating the energy of each model.

Since the main object of study in this paper is the SiCp/Al interface, the interface model needs to be constructed first. The different surfaces of SiC and Al are selected before constructing the interface model, and the (100), (110), (111), and (211) surface energies of Al are calculated using Equation (1) [26], and the (001), (011), (111), and (211) surface energies of SiC are calculated using Equation (2) [27].

(1) $$E_{surf}=\frac{1}{2A_{surface}}\left(E_{slab}^{total}-N_{Al}\mu_{i}\right)$$

(2) $$\sigma_{SiC}=\frac{1}{2A_{surface}}\left(E_{slab}-N_{Si}\mu_{Si}^{Slab}-N_{C}\mu_{C}^{slab}\right)$$

Here, *A_surface_* is the surface area; $E_{slab}^{total}\;$ is the surface energy; *N_Al_*, *N_Si_*, and *N_C_* are the total numbers of atoms; and *μ_Al_*, *μ_Si_*, and *μ_C_* are the atomic chemical potentials.

In addition, in order to shorten the calculation time, we calculate the interfacial adhesion work for both Si-top and C-top configurations separately, and the interfacial adhesion work *W_ad_* is calculated as follows [28]:

(3) $$W_{ad}=\frac{E_{Al}+E_{SiC}-E_{Al/SiC}\left(x\right)}{A}$$

*E_Al_* and *E_SiC_* are the energies of Al and SiC films optimized in the SiCp/Al interface, respectively. *E_Al/SiC_*(*x*) represents the energy after optimization of the SiCp/Al interface structure. A indicates the interface area of the SiCp/Al interface.

Both the surfaces of SiC and Al as well as the optimal interfacial distance were selected based on the calculation of surface energy and interfacial adhesion work, and the added alloying elements were placed at the interface and inside the Al matrix, respectively, and the interface segregation energy and interface binding energy of the model were calculated using Equation (4) [29] and Equation (5) [30].

(4) $$E_{segregation}=E_{interface}-E_{inside}$$

(5) $$E_{binding}=E_{interface}-E_{SiC}-E_{basis}$$

Here, *E_segregation_* is the interface segregation energy of the model after alloying elements are added, *E_binding_* is the interface binding energy of the model after alloying elements are added, *E_interface_* is the total energy of the model when alloying elements are located at the SiCp/Al interface, and *E_inside_* is the total energy of the system when alloying elements are located inside the model. *E_SiC_* is the energy of SiC reinforcement phase and *E_basis_* is the total energy of Al matrix part after adding alloying elements.

Due to the long time period required for first-principles calculation, it takes a long time to realize the screening of more than 100 alloying elements, so the machine learning method is used in this paper to accelerate the first-principles calculation and shorten the calculation time.

### 2.2. Machine-Learning-Accelerated First-Principle Computations

Due to the long time period required for first-principles calculation, it takes a long time to realize the screening of more than one hundred alloying elements, so in this paper, we use the machine learning method to accelerate the first-principles calculation and shorten the calculation time. The idea of machine learning to accelerate the calculation of the first principle is mainly composed of three parts. (1) The calculation of the nature of the model occurs based on the first-principle method. (2) The results of the first-principles calculation are screened and organized and the output part of the machine learning dataset can be initially obtained. We can use feature engineering to select the input features for machine learning, design the machine learning model by combining the existing inputs and outputs, and carry out the next prediction of the model with the best fit and the smallest error. (3) We use the best machine learning model for forward prediction to screen out the alloy elements that meet the requirements. The method of accelerating the first-principles calculation through machine learning makes the first-principles calculation time plummet from thousands of hours to tens of seconds, which greatly improves the calculation efficiency. Machine learning can not only screen alloy elements in the forward direction but also carry out reverse prediction. When a target value is given, the trained machine learning model can output the element characteristics corresponding to the value and the target element can be obtained directly.

In this paper, six machine learning methods, namely Support Vector Machine (SVM), Artificial Neural Network (ANN), BP Neural Network (BPNN), Radial Basis Neural Network (RBF), Integration Algorithm (ENS), and Random Forest (RF) methods, are used to train interface segregation energy and interface binding energy of alloying elements in interface models. SVM is a classification model that is essentially a linear classifier with the largest feature-space spacing. SVM has strong generalization ability and no local minimum problem. It is an optimization algorithm for solving convex quadratic programming [31]. ANN is a machine learning algorithm that simulates the connection and information transfer between human neurons, which are composed of a large number of neurons and the connections between them [32]. An input layer, an output layer, and several hidden layers constitute an artificial neural network, in which the input layer is responsible for receiving signals, the hidden layer is responsible for data decomposition and processing, and the final result is outputted after integration by the output layer [33]. Artificial neural network is an important machine learning algorithm that learns and trains by establishing complex connection relationships and realizes the processing and prediction of complex nonlinear mapping between input and output. ANN has broad development prospects in pattern recognition, automatic control, artificial intelligence, and other fields [34]. BPNN is an artificial neural network based on error backpropagation that is widely used to solve problems such as classification, regression, and data mining. The calculation accuracy of BPNN is high, but the calculation time is long, and it is easy to overfit [35]. RBF is a neural network using RBF as the activation function that has the advantages of simple training and fast convergence, but its interpretation is poor, and it cannot work when the data are insufficient. Therefore, RBF is mainly used in the field of function approximation [36]. Ensemble is an algorithm that accomplishes a learning task by building and combining multiple learners. The method of integrated learning selected in this paper is Stacking. The method is used to combine other models by training a model. Ensemble learning algorithms can solve many problems such as feature selection, image processing, transfer learning, etc. Ensemble can improve the accuracy and stability of the model when dealing with complex problems, but it is not suitable for small-scale datasets [37]. RF is a kind of integrated learning built on the basis of decision trees. Multiple decision trees are used for calculation, and then the output of multiple decision trees is integrated to get the output result [38]. RF not only has the advantages of decision trees but also prevents overfitting [39]. By comparing R^2^ and MSE of several machine learning models, this paper successfully selects the machine learning model with the best performance and generalization ability. The selected model can predict the interface segregation energy and interface binding energy of other elements in the periodic table, complete the screening of alloying elements, and achieve the goal of accelerating the first-principles calculation.

## 3. Results

### 3.1. First-Principles Calculation Results

The results of the surface energy calculations are shown in Table 1; the surface energy of Al (111) is the lowest because Al belongs to a face-centered cubic structure and (111) is the surface with the highest density of Al, and so Al (111) is chosen. The surface energy of SiC (001) surface is the lowest, the selected 4H-SiC belongs to a hexagonal crystal system, and the surface of (001) is the surface with the highest density as well, and so the surface of SiC (001) is chosen to construct the interface model.

**Figure 1:**
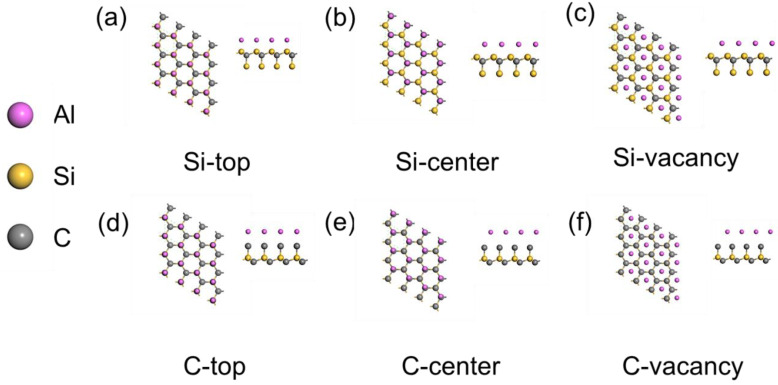
SiC (001)/Al (111) interfacial atomic structure: (**a**) Si-top; (**b**) Si-center; (**c**) Si-vacancy; (**d**) C-top; (**e**) C-center; (**f**) C-vacancy.

In order to reduce the calculation time of the first principle, this paper carries out the surface convergence calculations for Al and SiC’s. It is found that when the number of atomic layers of Al is greater than or equal to four, its surface energy almost no longer changes; when the number of atomic layers of SiC is greater than or equal to eight, its surface energy tends to be unchanged. Therefore, the Al atoms of the constructed interface model are in four layers and the SiC atoms are in eight layers. Since the surface of SiC has two structures, a C-terminal and Si-terminal, it constitutes an interface model with Al with six different structures as shown in Figure 1, namely the Si-top, Si-center, Si-vacancy, C-top, C-center, and C-vacancy. According to the results of existing studies, the structures of the Si-top and C-top are the most widely used and stable under realistic conditions. Therefore, in this paper, the total energy of the interface model is calculated for Si-top and C-top structures, and it is found that the total energy of the Si-top is lower than that of the C-top and that the structure with Si as the top is more stable than that with the C-top.

**Figure 2:**
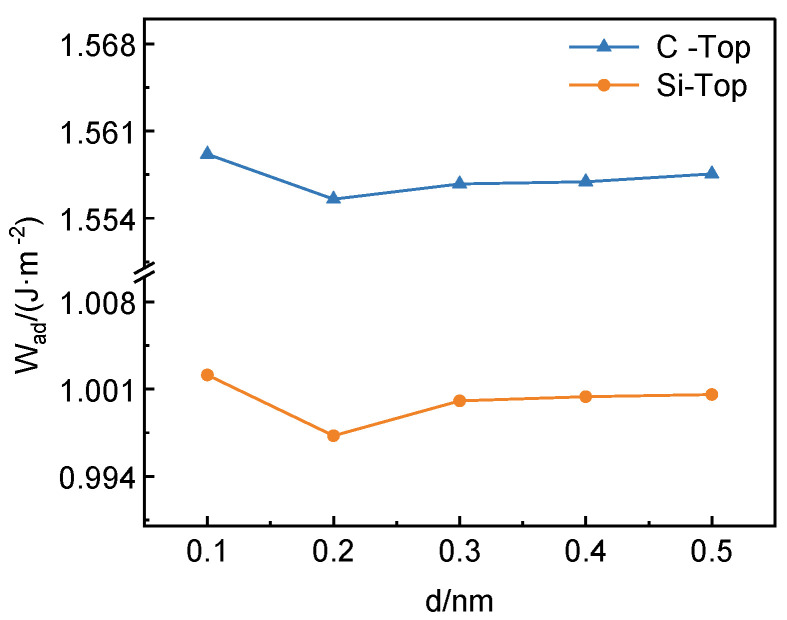
The relationship between SiC (001)/Al (111) interface separation work and interface distance.

According to the formula of interfacial adhesion work, the model energy values of different interfacial distances can be calculated and the optimal interfacial spacing can be screened out. The interfacial spacings of 0.1 nm, 0.2 nm, 0.3 nm, 0.4 nm, and 0.5 nm were selected, and the relationship between the interfacial distance and the interfacial adhesion work is shown in Figure 2. The interfacial adhesion work decreases with the increase in interfacial spacing and then increases with the increase in interfacial distance for both Si-top and C-top structures, and the interfacial adhesion work reaches the lowest value when the interfacial spacing is 0.2 nm.

**Figure 3:**
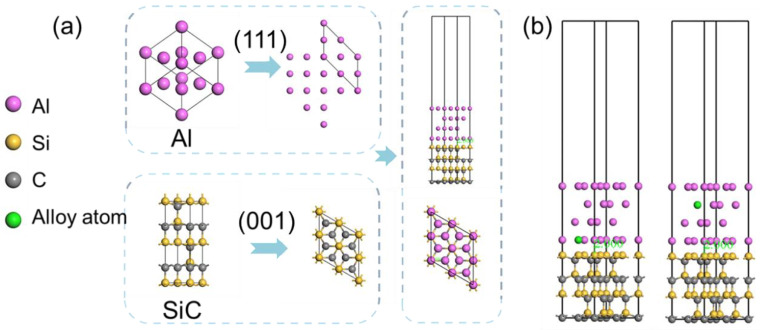
(**a**) SiCp/Al interface model construction process. (**b**) Initial structural model.

In summary, as shown in Figure 3a, the Al (111) plane and SiC (100) plane with Si as the top were selected to construct an interface model with an interface distance of 0.2 nm and a vacuum layer thickness of 20 Å. The SiCp/Al interface model was established by placing the added alloying elements at the interface and inside the Al matrix. Subsequently, 25 typical alloying elements were chosen from the periodic table, encompassing metallic, nonmetallic, semiconducting, and rare earth elements. The chosen alloying elements are evenly distributed across the periodic table, ensuring the randomness of the dataset sampling and enhancing the accuracy of the machine-learning prediction results. As depicted in Figure 3b, 25 standard alloying elements were positioned within the Al matrix and at the SiCp/Al interface.

**Figure 4:**
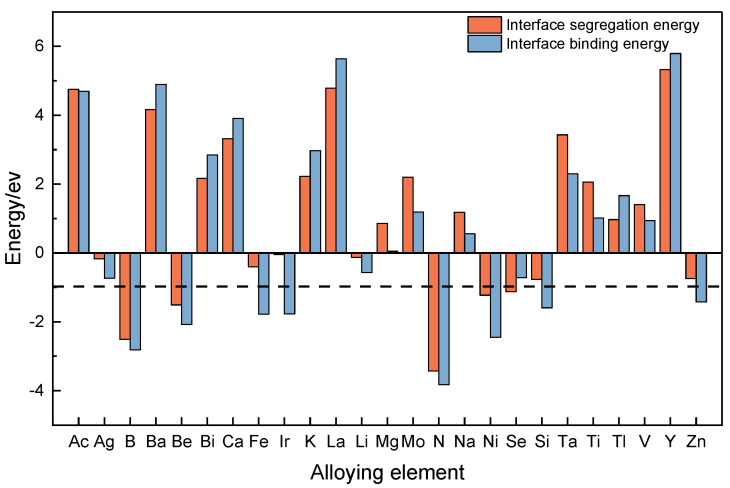
Calculations for typical alloying elements.

We utilize Equations (4) and (5) to compute the interface segregation energy and interface binding energy for the interface model incorporating additional alloying elements, respectively. The results of the calculations are presented in Figure 4. The red portion represents the interface segregation energy of the model while the blue portion represents the interface binding energy of the model. The value of the dashed line corresponds to the interface binding energy of the model at the SiCp/Al interface. Figure 4 illustrates that eleven out of the twenty-five typical alloying elements exhibit interfacial partial cohesion energies below 0, while eight demonstrate interface binding energies lower than those of the system without added alloying elements. Consequently, the alloying elements determined through first principles are deemed to be significant, and the findings are structured to form the output component of the machine learning dataset.

### 3.2. Database Establishment and Selection of Feature Values

The output portion of the machine learning dataset is obtained after the first-nature-principle computation, and the input features of the dataset are determined using feature engineering below. Based on the nature of interface binding energy and interface segregation energy, we selected 24 features that can express the nature of alloying elements. Next, we performed dimensionality reduction on the existing features using a Principal Component Analysis (PCA) of feature engineering. PCA is the process of discarding some of the original data or creating some new data, thus transforming the high-dimensional data into low-dimensional data [40]. PCA is based on the main idea of finding the largest direction of data change, so it is possible to reduce the dimensionality while still retaining the most significant components. Therefore, the use of PCA can greatly reduce the cost of computation and storage under the condition of ensuring the accuracy of the calculation, and it can also filter meaningless data and improve the prediction accuracy of machine learning [41]. PCA is mainly divided into the following parts: (1) data normalization; (2) covariance calculation; (3) calculating eigenvalues and eigenvectors; and (4) calculating each principal component and its contribution rate. This paper uses the Random Forest algorithm to realize the principal component analysis of the dataset. The main idea is to judge how much contribution each feature makes to each tree in the Random Forest and then take the average value to compare the contribution between features. Finally, a feature is selected according to the importance of the feature in order to achieve the dimensionality reduction of the feature.

**Figure 5:**
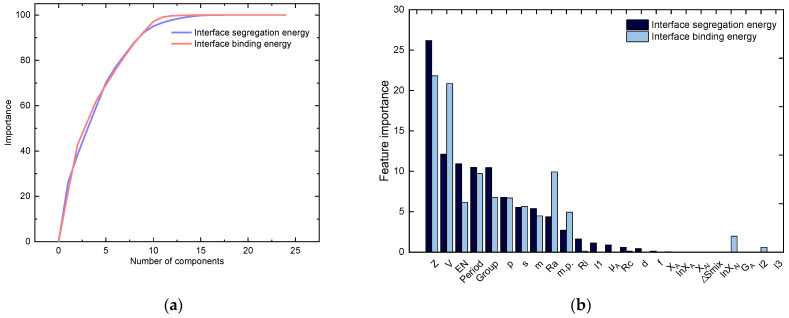
Results of PCA: (**a**) effect of the number of features on the result; (**b**) importance of each feature for predicting the output of interface segregation energy and interface binding energy.

The PCA calculation results are shown in Figure 5. From Figure 5a, it can be found that when the interface segregation energy and the number of input features combined with the interface are greater than or equal to 10, the feature contribution rate reaches 95%. In Figure 5b, the importance of each feature can be obtained. The top ten features selected by ranking their importance are the features selected by PCA dimensionality reduction. In this paper, the interface binding energy and interface segregation energy are screened. The characteristics of both are atomic number, period, main group, electronegativity, atomic volume, melting point, relative atomic mass, atomic radius, electron configuration s, and electron configuration p. The selected features are the input part of the dataset, and the calculated interface segregation energy and interface binding energy are the output part of the dataset. At this point, the construction of the dataset and the calculation of the feature screening part are completed.

### 3.3. Machine Learning Model Construction and Selection

After the dataset is established, we need to build the model of machine learning and select the most suitable model to make it accelerate the first-principles calculation. Therefore, in this paper, we have chosen six methods of machine learning, the SVM, BPNN, ANN, RBF, RF, and Ensemble methods, to build models and train the existing dataset. In order to ensure the accuracy of the machine learning results, we divided the dataset into two parts: the training set and the test set. Among them, to ensure the learning effect of the model, the training set comprises 80% and the test set comprises 20%.

There are many indicators for evaluating the goodness of machine learning models, such as the R^2^, Adjusted-R^2^, MSE, RMSE, MAE, and MAPE. In this paper, we have chosen R^2^ and MSE as the evaluation indicators of regression models. The coefficient of determination (R^2^) reflects the degree of model fit, and the range of R^2^ is from 0 to 1. The closer its value is to 1, the stronger the explanatory power of the equation is, and the better the model in question fits the data. The mean square error (MSE) is the square of the difference between the true value and the predicted value, and then the average of the summation, which is generally used to detect the deviation between the predicted and true values of the model. Therefore, when faced with the selection of a machine learning model, it is important to choose one that has a good fit, i.e., a large R^2^, and one that has a small deviation between the predicted and true values, i.e., a small MSE. Considering only one of the cases will affect the subsequent prediction accuracy. For example, if the R^2^ of the model is large but the MSE is also large, overfitting will occur and the reliability of the prediction results will not be high; on the contrary, if the R^2^ of the model is very small and the MSE is also small, this case indicates that the machine learning model does not have a good fit to the dataset and that it cannot explain the laws of the dataset. Therefore, we trained the dataset constructed in the previous section with six machine learning methods and obtained the R^2^ and MSE values of the interface segregation energy and interface binding energy machine learning models, respectively.

**Figure 6:**
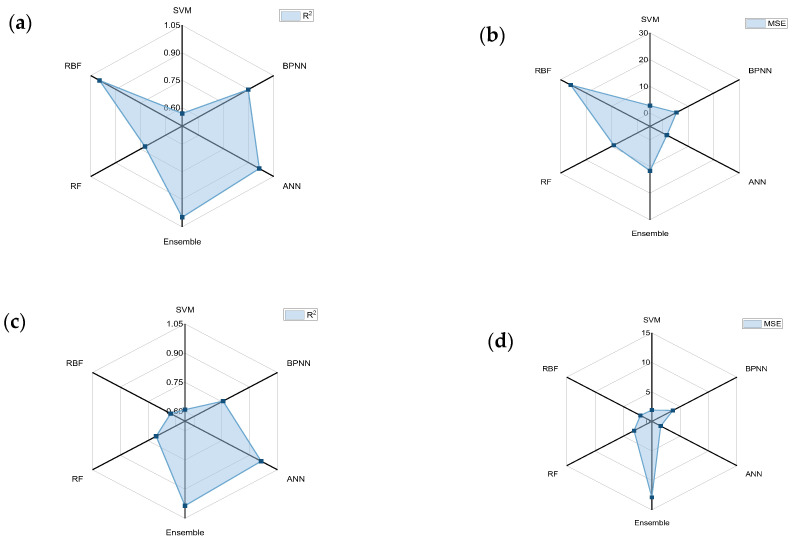
Accuracy of different models: (**a**) R^2^ of interface binding energy; (**b**) MSE of interface binding energy; (**c**) R^2^ of interfacial segregation energy; (**d**) MSE of interfacial segregation energy.

The R^2^ and MSE calculations for each model are shown in Figure 6a, which represents the R^2^ value of the interface binding energy, and the R^2^ is sorted thus: RBF > ANN > Ensemble > BPNN > RF > SVM. Figure 6b represents the MSE of the interface binding energy, and the MSE is sorted thus: RBF > RF > Ensemble > BPNN > SVM > ANN. It is found that although the R^2^ value of the RBF is very large, its prediction value also has a large error with the true value being very large but the error between its predicted and true values also being large. Then, comparing the MSE of its training set and test set, it is found that the error of the training set is only 2 × 10^−5^ while the error of the test set is 8.95. Therefore, the RBF has an overfitting phenomenon when training on the dataset, and it cannot be used for prediction in machine learning. Comparing the training results of the ANN, not only is the value of R^2^ larger but also the value of MSE is the smallest, and the difference between the error values of its training set and test set is not much, which means it can be used as a machine learning model for this dataset. After comparison, it is found that the rest of the machine learning methods are good, but the results are still not accurate enough when compared with the ANN algorithm. Figure 6c,d represent the R^2^ and MSE of the interface segregation energy. It can be found that in the process of calculating the interface segregation energy, the Ensemble method also suffers from the overfitting problem in the calculation of the interface binding energy, and the remaining four methods, namely the RBF, the BPNN, the RF, and the SVM, have a lower degree of fit and larger errors. The ANN is also the optimal choice for the machine learning model to predict the interface segregation energy.

The selected artificial neural network (ANN) machine learning model for predicting interface binding energy and interface segregation energy comprises an input layer, an output layer, and a hidden layer with ten neurons. The model was trained using the Levenberg–Marquardt (L-M) algorithm with a randomized division of the data, and the training process involved seven iterations. The number of neurons in the hidden layer was modified to decrease computational time while preserving computational accuracy. A reduction in the number of neurons leads to both an increase in the mean squared error (MSE) for both the test and training sets as well as a decrease in the R^2^. Conversely, an increase in the number of neurons results in higher computational requirements and longer processing times. Thus, the presently chosen artificial neural network (ANN) model is deemed the most appropriate machine learning model for the dataset of DFT calculation results. The ten feature descriptors, including the atomic number, period, main group, atomic radius, melting point, relative atomic mass, electron configurations s and p, atomic volume, and electronegativity, are chosen as input variables for the input layer. Following training in the ten hidden layers, these descriptors are then outputted in the output layer, which in turn outputs the interface segregation energy or the interface binding energy. Machine learning has been employed to predict interface segregation energy and interface binding energy in order to expedite first-principles calculations.

**Figure 7:**
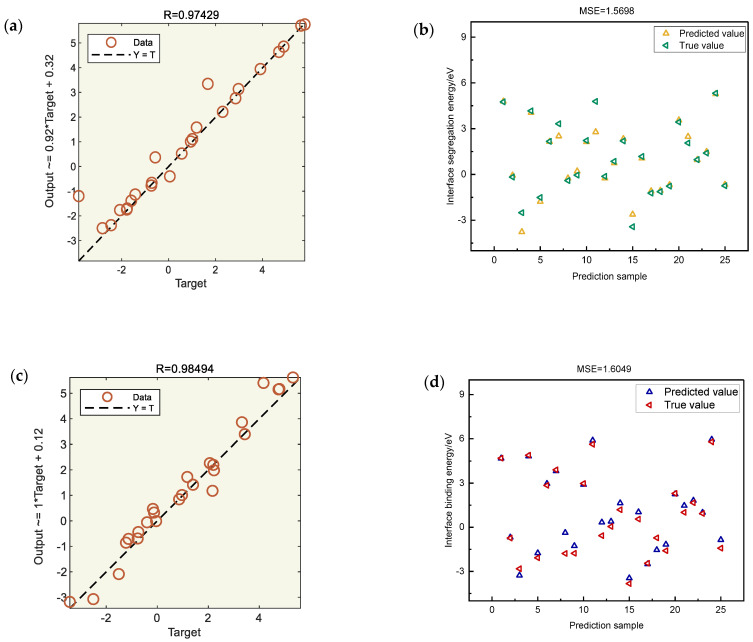
Results of the ANN model: (**a**,**b**) R and MSE of interfacial segregation energy; (**c**,**d**) R and MSE of interface binding energy.

The datasets of interface segregation energy and interface binding energy of the machine learning model built above are used for training, and their R^2^ values and MSE values are obtained. It can be seen from Figure 7 that the R^2^ value in the machine learning model, whether it is of the interface segregation energy or the interface binding energy, is greater than 0.95, and the MSE value is less than 2. Therefore, the model fully meets the requirements of the machine model, and the next step can be taken to predict the remaining elements of the element cycle.

### 3.4. Screening of Alloying Elements for Interface Modulation

A trained artificial neural network (ANN) model was employed to forecast the interface segregation energy and interface binding energy for an interfacial model with the inclusion of the remaining elements. The range of elements screened encompasses all elements of the periodic table with the exception of noble gases, heavy elements, and certain actinides. The noble gases were excluded due to their lack of reactivity with other elements and their primary use as protective atmospheres in metallic materials. Heavy metals and actinides were omitted from the screening for two primary reasons. Firstly, the existing studies have incomplete data on the properties of these elements. Secondly, accurate results are challenging to compute using first-nature-principle calculations due to numerous errors. Machine learning was employed to expedite first-principles calculations for the screening of the remaining 89 alloying elements using a dataset of 25 alloying elements. This involved the implementation of accelerated first-principles calculations with a mini-sample machine learning model.

We used the dataset calculated using the DFT to train the machine learning model, then used the model to predict the interface segregation energy and interface binding energy; finally, we found that according to the prediction of the two screened elements, to take the same portion of the screened alloying elements can not only inhibit the SiCp/Al interfacial reaction to reduce the Al_4_C_3_ phase but also enhance the interfacial bonding ability of SiCp/Al. For the screening of interface segregation energy, the main purpose is to screen out the elements with negative interface segregation energy. According to the formula of interface segregation energy, the presence of negative interface segregation energy indicates that the energy of the alloying element at the interface is less than that of the alloying element inside the matrix, and the lower the energy is, the more the element tends to exist at the interface rather than inside, which can inhibit the interfacial reaction of SiCp/Al. For the screening of the interface binding energy, the interface binding energy of the model with the added alloying element was compared with that of the unadded model. The interface binding energy of the SiCp/Al interface model without the alloying element was calculated to be −0.97401 eV as per the first-nature principle, and if the value of the interface binding energy of the interface model was less than −0.97401 eV after the addition of the alloying element, it would mean that the addition of the alloying element had improved the strength of interfacial bonding.

**Figure 8:**
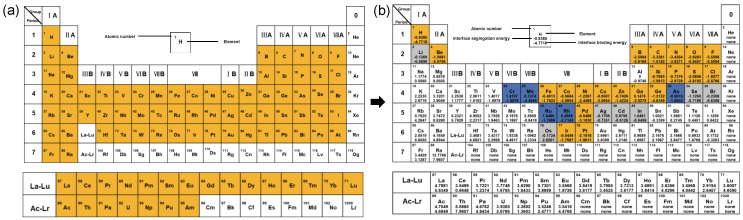
Matrix alloy element screening process: (**a**) screening range for alloying elements; (**b**) alloying elements screened.

Figure 8 shows the screening process of the alloying elements. The alloying elements in the yellow part of the figure are easy to polarize at the interface and enhance the interfacial bonding, the alloying elements in the blue part of the figure can improve the bonding force between the SiCp/Al interface, and the alloying elements in the gray part of the figure are inclined to polarize to the interface and play the role of inhibiting the interfacial reaction. There are 28 combined elements screened by interface segregation energy and 26 alloying elements screened by interface binding energy. There are 21 alloying elements that can both inhibit interfacial reactions and enhance SiCp/Al interfacial bonding. Among them, S, P, O, and N are considered impurity elements in metal materials and cannot be added to the aluminum matrix; C will react with Al to increase the Al_4_C_3_ phase, which makes the material performance plasticity reduced and strength weakened; Cl and F as halogenated elements will have a corrosive effect on the metal materials, so they also cannot be added to the metal materials; Be is toxic and is rarely used in metal materials; and Pd, Ir, and Pt are heavy-metal elements, the cost is too high, and the current application is not too widespread. Adding H in the aluminum matrix causes hydrogen embrittlement and affects the material performance. Therefore, the following nine alloying elements were finally selected: B, Si, Fe, Co, Ni, Cu, Zn, Ga, and Ge.

**Figure 9:**
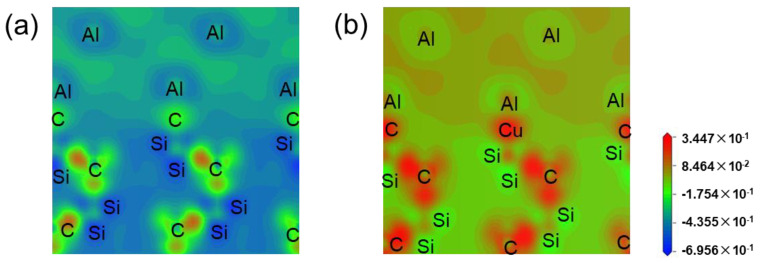
Differential charge density for different interface models: (**a**) SiCp/Al; (**b**) SiCp/Al-Cu.

**Figure 10:**
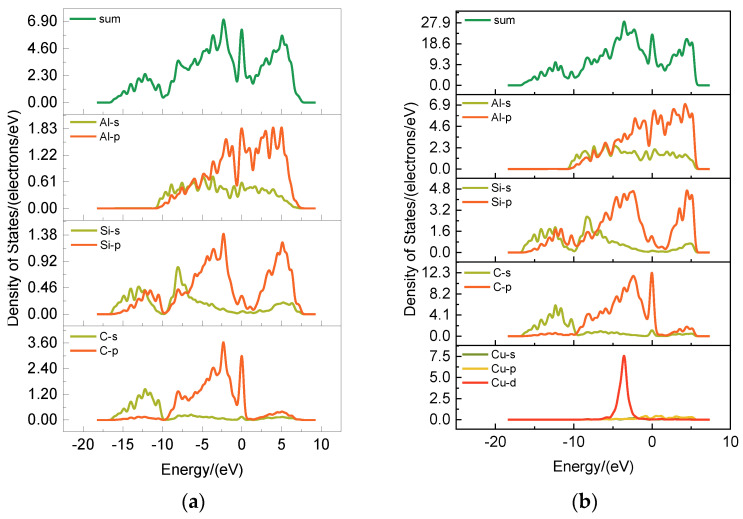
Density of states for different interface models: (**a**) SiCp/Al; (**b**) SiCp/Al-Cu.

Nine target alloying elements were screened out after the calculation of first-nature principles and machine learning. We choose one of them, Cu, to explain the effect of this alloying element on the electronic structure of the interface from an atomic point of view based on the first-nature principles. Shown in Figure 9 are the differential charge density maps of SiCp/Al and SiCp/Al-Cu systems. The differential charge density was analyzed, and it was found that the addition of Cu atoms to the system caused a great change in the electronic structure of the material system due to the doping of the alloying atoms. When no Cu atoms were added, comparing the similar C and Al atoms, C atoms had a larger negative charge, Al atoms had a larger positive charge, and there were strong ionic-bonding interactions between them, making the material plasticity and toughness deteriorate. Adding Cu changed the above C and Al atoms’ differential charge density distribution, ionic-bonding specific gravity became smaller, and there was more embodiment of the metallic nature, which is conducive to the improvement of composite material brittleness. The fractional-wave densities of states for the SiCp/Al and SiCp/Al-Cu systems are shown in Figure 10. The increase in the values of the densities of states after the addition of the Cu atoms indicates that the electrons cross the Fermi surface and jump from the valence state to the conduction band and that there is a larger interaction between the SiC and Al atoms and the Cu, which further indicates that the system is more metallic. The orbital coupling of the 3d orbitals of Cu with the 2p orbitals of C, Si, and Al, as well as with the 2s orbital of Al, indicates that Cu interacts with all of these atoms. In addition, the orbital coupling between C and Al is weakened by the addition of Cu atoms, which implies that the introduction of Cu inhibits the interfacial reaction to some extent. Therefore, the addition of interface-regulating alloying elements can improve the material properties, control the interfacial reaction, and enhance the interfacial bonding to a certain extent.

## 4. Conclusions

In this paper, we have presented a machine-learning-based approach that significantly enhances the efficiency of screening interfacially modified elements through accelerated first-principles calculations. The results of first-principles calculations were used as a machine learning dataset to select the best-performing machine learning models to predict the remaining 89 alloying elements in the periodic table. The screening process identified 28 alloying elements based on interface segregation energy and 25 alloying elements based on interface binding energy. Further screening was carried out based on existing studies, and the following nine alloying elements were finally screened: B, Si, Fe, Co, Ni, Cu, Zn, Ga, and Ge. The results of the differential charge density analysis of the model of the SiCp/Al-Cu system using first-principles calculations confirm the enhancement of the metallicity of the system with the addition of alloying elements. The screened alloying elements not only inhibit interfacial reactions in SiCp/Al but also enhance metallic bonding, improve material toughness, and augment the interfacial bonding between the reinforcement and matrix, consequently increasing material strength. Overall, this paper demonstrates an improved screening efficiency for interface-modified alloying elements through the integration of machine learning and computational first-principles calculations.

## Figures and Tables

**Table 1:** Surface energy values of different surfaces of Al and SiC.

Al_surf_	E_surf_/(J·m^−2^)	SiC_surf_	E_surf_/(J·m^−2^)
(111)	0.83107594	(111)	3.83029199
(110)	0.97319305	(011)	3.22173635
(100)	0.95097756	(001)	2.96169004
(211)	1.06830391	(211)	4.21076767

## Data Availability

Data are contained within the article and Supplementary Materials.

## Supplementary Materials

The following supporting information can be downloaded at: https://www.mdpi.com/article/10.3390/ma17061322/s1, Table S1: Feature descriptor and its calculation formula.

## Appendix A

The four alloying elements Fe, Co, Ni, and Cu are considered to be magnetic. We calculated the model with spin polarization added and the model without spin polarization separately, and the results are shown in Figure A1. It can be seen that whether spin polarization is considered has little effect on the interface polarization and interface binding energy of the model, so the spin polarization of magnetic alloy elements was not involved in the calculation in this paper.

## References

[1] Tahani M., Postek E., Sadowski T. (2023). Investigating the Influence of Diffusion on the Cohesive Zone Model of the SiC/Al Composite Interface. Mloecules.

[2] Ferraris M., Gili F., Lizarralde X., Igartua A., Mendoza G., Blugan G., Gorjan L., Casalegno V. (2022). SiC particle reinforced Al matrix composites brazed on aluminum body for lightweight wear resistant brakes. Ceram. Int..

[3] Gorbatyuk S.M., Pashkov A.N., Morozova I.G., Chicheneva O.N. (2021). Technologies for applying Ni-Au coatings to heat sinks of SiC-Al metal matrix composite material. Mater. Today Proc..

[4] Tiwari A., Agarwal M., Dixit M., Agrawal P. (2023). Impact of SiC Addition on the Metallurgical and Microstructural Properties of Gravity Die Cast Composite for AA6061 Matrix. Trans. Indian Inst. Met..

[5] Ahmadi M., Ansari R., Hassanzadeh-Aghdam M.K. (2023). Micromechanical finite element analysis of Young’s modulus, yield strength and thermal expansion coefficient of nano-sized ceramic particle/metal matrix nanocomposites. J. Braz. Soc. Mech. Sci. Eng..

[6] Shin S., Cho S., Lee D., Kim Y., Lee S.B., Lee S.K., Jo I. (2019). Microstructural Evolution and Strengthening Mechanism of SiC/Al Composites Fabricated by a Liquid-Pressing Process and Heat Treatment. Materials.

[7] Mi G., Xiang Y., Wang C., Xiong L., Ouyang Q. (2023). Microstructure and mechanical properties of SiCp/Al composite fabricated by concurrent wire-powder feeding laser deposition. J. Mater. Res. Technol..

[8] Hamasuna K., Iwamoto C., Satonaka S., Nishida M., Tomoshige R., Fujita M. (2007). Development of resistance welding for silicon carbide. Mater. Trans..

[9] Tahani M., Postek E., Motevalizadeh L., Sadowski T. (2023). Effect of Vacancy Defect Content on the Interdiffusion of Cubic and Hexagonal SiC/Al Interfaces: A Molecular Dynamics Study. Molecules.

[10] Pech-Canul M.I., Katz R.N., Makhlouf M.M., Pickard S. (2000). The role of silicon in wetting and pressureless infiltration of SiCp preforms by aluminum alloys. J. Mater. Sci..

[11] Kajikawa Y., Nukami T., Flemings M.C. (1995). Pressureless infiltration of aluminum metal-matrix composites. Metall. Mater. Trans. A.

[12] Lepen E., Le Pen E., Baptiste D., Hug G. (2002). Multi-scale fatigue behaviour modelling of Al-Al2O3 short fibre composites. Int. J. Fatigue.

[13] Fouret C., Degallaix S. (2002). Experimental and numerical study of the low-cycle fatigue behaviour of a cast metal matrix composite Al-SiC_p_. Int. J. Fatigue.

[14] Kumar V., Sharma V. (2019). Effects of *SiC*, Al_2_O_3_, and ZrO_2_ particles on the LBMed characteristics of Al/*SiC*, Al/Al_2_O_3_, and Al/ZrO_2_ MMCs prepared by stir casting process. Part. Sci. Technol..

[15] Jojith R., Radhika N. (2021). Reciprocal dry sliding wear of SiCp/Al-7Si-0.3 Mg functionally graded composites: Influence of T6 treatment and process parameters. Ceram. Int..

[16] Chong S.-Y., Atkinson H.-V., Jones H. (1993). Effect of ceramic particle-size, melt superheat, impurities and alloy conditions on threshold pressure for infiltration of SiC powder compacts by aluminum-based melts. Mater. Sci. Eng. A-Struct. Mater. Prop. Microstruct. Process..

[17] Ocelík V., Matthews D., De Hosson J. (2005). Sliding wear resistance of metal matrix composite layers prepared by high power laser. Surf. Coat. Technol..

[18] Prusov E.S., Kechin V.A., Deev V.B., Shurkin P.K. (2022). Thermodynamics of the Effect of Alloying of Phase Formation during Crystallization of Aluminum Matrix Composites with Exogenous Reinforcement. Russ. J. Non-Ferr. Met..

[19] Munir K.S., Li Y., Liang D., Qian M., Xu W., Wen C. (2015). Effect of dispersion method on the deterioration, interfacial interactions and re-agglomeration of carbon nanotubes in titanium metal matrix composites. Mater. Des..

[20] Fukumoto A. (1996). First-principles calculations of p-type impurities in cubic SiC. Phys. Rev. B.

[21] Matsushima N., Jun Y. (2019). First-principles X-ray photoelectron spectroscopy binding energy shift calculation for boron and aluminum defects in 3C-silicon carbide. Jpn. J. Appl. Phys..

[22] Miyazato I., Yuzuru T., Keisuke T. (2018). Accelerating the discovery of hidden two-dimensional magnets using machine learning and first principle calculations. J. Phys. Condens. Matter.

[23] Artrith N., Urban A., Ceder G. (2018). Constructing first-principles phase diagrams of amorphous Li_x_Si using machine-learning-assisted sampling with an evolutionary algorithm. J. Chem. Phys..

[24] Cytter Y., Rabani E., Neuhauser D., Preising M., Redmer R., Baer R. (2019). Transition to metallization in warm dense helium-hydrogen mixtures using stochastic density functional theory within the Kubo-Greenwood formalism. Phys. Rev. B.

[25] Rahmani-Ivriq N., Kordbacheh A.A. (2021). Rectifying and spin filtering behavior of aluminum doped silicon carbide nanoribbons: The first principles study. J. Phys. D-Appl. Phys..

[26] Wang Y., Li M., Peng P., Gao H., Wang J., Sun B. (2022). Preferred orientation at the Al/graphene interface: First-principles calculations and experimental observation. J. Alloys Compd..

[27] Zou A.-H., Zhou X.-L., Kang Z.-B., Rao Y.-H., Wu K.-Y. (2019). Alloy Elements on SiC/Al Interface: A First-principle and Experimental Study. J. Inorg. Mater..

[28] Li Y.F., Xiao B., Wang G.L., Sun L., Zheng Q.L., Liu Z.W., Gao Y.M. (2018). Revealing the novel fracture mechanism of the interfaces of TiB2/Fe composite from a first principles investigation. Acta Mater..

[29] Xie Y.P., Zhao S.J. (2012). First principles study of Al and Ni segregation to the α-Fe/Cu (100) coherent interface and their effects on the interfacial cohesion. Comput. Mater. Sci..

[30] Liu G.-L., Guo Y.-F., Li R.-D. (2007). Electronic theory of interface characteristics of ZA27/CNT. Acta Phys. Sin..

[31] Hasan S., Kordijazi A., Rohatgi P.K., Nosonovsky M. (2021). Triboinformatic modeling of dry friction and wear of aluminum base alloys using machine learning algorithms. Tribol. Int..

[32] Chaluvaraju B.V., Afzal A., Vinnik D.A., Kaladgi A.R., Alamri S., Tirth V. (2021). Mechanical and Corrosion Studies of Friction Stir Welded Nano Al_2_O_3_ Reinforced Al-Mg Matrix Composites: RSM-ANN Modelling Approach. Symmetry.

[33] Majumdar A., Jindal A., Arora S., Bajya M. (2022). Hybrid Neuro-Genetic Machine Learning Models for the Engineering of Ring-spun Cotton Yarns. J. Nat. Fibers.

[34] Sheikh-Ahmad J., Twomey J. (2007). ANN constitutive model for high strain-rate deformation of Al 7075-T6. J. Mater. Process. Technol..

[35] Abbas A.T., Sharma N., Anwar S., Luqman M., Tomaz I., Hegab H. (2020). Multi-Response Optimization in High-Speed Machining of Ti-6Al-4V Using TOPSIS-Fuzzy Integrated Approach. Materials.

[36] Kumar B.G., Kumar R.L., Ramalingam V.V., Viswanath J.K., Harikeshava R., Padmanaban R. (2022). Corrosion and Tribological Characteristics of Friction Stir Processed Aluminium Alloy AA5052. Trans. Marit. Sci. Toms.

[37] Ahmad A., Ostrowski K.A., Maślak M., Farooq F., Mehmood I., Nafees A. (2021). Comparative Study of Supervised Machine Learning Algorithms for Predicting the Compressive Strength of Concrete at High Temperature. Materials.

[38] Moayedi H., Bui D.T., Kalantar B., Foong L.K. (2019). Machine-Learning-Based Classification Approaches toward Recognizing Slope Stability Failure. Appl. Sci..

[39] Dinaharan I., Palanivel R., Murugan N., Laubscher R.F. (2020). Predicting the wear rate of AA6082 aluminum surface composites produced by friction stir processing via artificial neural network. Multidiscip. Model. Mater. Struct..

[40] Koo S., Shin D., Kim C. (2021). Application of Principal Component Analysis Approach to Predict Shear Strength of Reinforced Concrete Beams with Stirrups. Materials.

[41] Ali N.M., Mustafah Y.M., Rashid N.M. (2013). Performance analysis of robust road sign identification. Conf. Ser. Mater. Sci. Eng..

