# Supplementary material for: Accelerated First-Principles Calculations Based on Machine Learning for Interfacial Modification Element Screening of SiCp/Al Composites
Source: Materials (Basel). 2024 Mar 13;17(6):1322. doi: 10.3390/ma17061322 (PMC10972090; doi:10.3390/ma17061322)
Supplement: Supplementary file 1 [file materials-17-01322-s001.zip › materials-2869181-supplementary.pdf]

materials

MDPI

Supplementary materials

This table shows the 24 features selected by relying on relevant literature and thermodynamic equation derivation. Some of the eigenvalues were obtained by searching the relevant literature and the rest of the eigenvalues were obtained by calculating the formulas in the table.

Table S1. Feature descriptor and its calculation formula.

Feature	Symbol	Formula
Atomic number	Z	/
Period	/	/
Group	/	/
Electronegativity	EN	/
Atomic volume	V	$V=m/\rho$
Melting point	m.p.	/
Correlated atomic mass	m	/
Atomic radius	Ra	/
Ionic radius	Ri	/
Covalent radius	Rc	/
Electron configuration s	s	/
Electron configuration p	p	/
Electron configuration d	d	/
Electron configuration f	f	/
Mole fraction of alloying elements	X_A	$X_A= m_A/(m_A+ m_{Al}+ m_{SiC})$
Logarithm of the mole fraction of alloying elements	$\ln X_A$	$\ln X_A= \ln X_A$
Mole fraction of the aluminum matrix	X_{Al}	$X_{Al}= m_{Al}/(m_A+ m_{Al}+m_{SiC})$
Logarithm of the mole fraction of the aluminum matrix	$\ln X_{Al}$	$\ln X_{Al}=\ln X_{Al}$
Entropy of mixing	ΔS_{mix}	$\Delta S_{mix}=-R(X_A\ln X_A+X_{Al}+\ln X_{Al})$
Gibbs free energy of the alloying element	G_A	$G_A=U-TS$
Hemical potential	μ_A	$\mu_A=G_A+RT\ln X_A$
First ionization energy	I1	/
Second ionization energy	I2	/
Third ionization energy	I3	/
